# Discovery of putative capsaicin biosynthetic genes by RNA-Seq and digital gene expression analysis of pepper

**DOI:** 10.1038/srep34121

**Published:** 2016-10-19

**Authors:** Zi-Xin Zhang, Shu-Niu Zhao, Gao-Feng Liu, Zu-Mei Huang, Zhen-Mu Cao, Shan-Han Cheng, Shi-Sen Lin

**Affiliations:** 1 Key Laboratory of Protection and Development Utilization, Tropical Crop Germplasm Resources (Hainan University), Ministry of Education, Haikou 570228, China; 2 Tropical Crops Genetic Resources Institute, Chinese Academy of Tropical Agricultural Sciences/Key Laboratory of Crop Gene Resources and Germplasm Enhancement in Southern China, Ministry of Agriculture, Danzhou 571737, China; 3 College of Horticulture and Landscape, Hainan University, Haikou 570228, China; 4 College of Horticulture, Nanjing Agricultural University, Nanjing 210095, China

## Abstract

The Indian pepper ‘Guijiangwang’ (*Capsicum frutescens L.*), one of the world’s hottest chili peppers, is rich in capsaicinoids. The accumulation of the alkaloid capsaicin and its analogs in the epidermal cells of the placenta contribute to the pungency of *Capsicum* fruits. To identify putative genes involved in capsaicin biosynthesis, RNA-Seq was used to analyze the pepper’s expression profiles over five developmental stages. Five cDNA libraries were constructed from the total RNA of placental tissue and sequenced using an Illumina HiSeq 2000. More than 19 million clean reads were obtained from each library, and greater than 50% of the reads were assignable to reference genes. Digital gene expression (DGE) profile analysis using Solexa sequencing was performed at five fruit developmental stages and resulted in the identification of 135 genes of known function; their expression patterns were compared to the capsaicin accumulation pattern. Ten genes of known function were identified as most likely to be involved in regulating capsaicin synthesis. Additionally, 20 new candidate genes were identified related to capsaicin synthesis. We use a combination of RNA-Seq and DGE analyses to contribute to the understanding of the biosynthetic regulatory mechanism(s) of secondary metabolites in a nonmodel plant and to identify candidate enzyme-encoding genes.

Capsaicinoids, which accumulate in the vesicles and vacuoles of epidermal cells of the placenta^1^, are responsible for the pungent taste of chili pepper (*Capsicum sp.*) fruits. Capsaicin and dihydrocapsaicin are the primary determinants capsaicinoid content of chili pepper fruits, accounting for 90% of the total content. As the source of their pungency, the concentration of capsaicinoids is the most important trait of peppers. Capsaicinoids are widely used in many scientific fields including the food, cosmetic, and pharmaceutical industries^2^. Thus, high capsaicinoid content is an important breeding goal for this crop plant. However, the limited material and dynamics of capsaicin synthesis complicate the genetics and gene discovery. Moreover, the molecular basis of capsaicin biosynthesis has not yet been explored. ‘Guijiaowang’ is a species of pepper that contains abundant capsaicinoids and, unlike other highly pungent pepper species, it displays obvious changes at different developmental stages. Therefore, we used this species to investigate capsaicin biosynthesis in pepper.

Capsaicinoids are synthesized via both the phenylpropanoid and the branched-fatty-acid pathways^3^. Numerous enzymes are involved in capsaicin biosynthesis, but many of these are not well characterized, and pathway regulation is not fully understood. Phenylalanine ammonia-lyase (PAL) is the first enzyme in the phenylpropanoid pathway, in which cinnamic acid is formed from phenylalanine^4^, followed by cinnamatc 4-hydroxylase (C4H), 4-Coumarate:Coenzyme A Ligase (4CL), hydroxycinnamoyl transferase (HCT), coumarate3-hydroxylase (C3H), caffeoyl-CoA 3-O-methyltransferase (CCoAOMT), and a putative aminotransferase (pAMT) to form vanillylamine. In the branched-fatty-acid synthesis pathway, the precursor 8-methyl-6-nonenoyl-CoA is produced via the fatty-acid synthesis cycle by a series of several enzymatic reactions^5^,^6^. In the end, vanillyamine and 8-methyl-6-nonenoyl-CoA, which are produced as two branched chains, are converted to capsaicin by acyl-transferase (AT)^7^. Capsaicinoid biosynthesis has long been of interest to researchers, and the pathway for the synthesis of all capsaicinoids has been proposed^7^. However, the regulation of the abundance of each analog in the fruit is not well understood. Over fifty gene families are involved in capsaicinoid biosynthesis^8^,^9^. Previous studies have indicated that the *Pun1* locus, which is required for the presence of capsaicinoids in pepper, encodes a putative acyltransferase^7^,^10^,^11^. Quantitatively inherited traits that control developmental processes in pepper are influenced by many genes with small effects^10^. Thus, their direct participation in the capsaicinoid biosynthesis pathway has not been demonstrated^12^.

A comparison of cDNA libraries prepared from the placenta of chili fruit indicated that *PAL, Ca4H, CCoAOMT, Kas,* and *pAMT* are differentially expressed. Moreover, their expression at the transcriptional level in the placenta is positively correlated with the degree of pungency in the chili pepper^13^. Biochemical analyses of placental tissues have shown that the independent silencing of *CCoAOMT, Kas* and *pAMT* can dramatically reduce capsaicinoid content in fruits^12^. Previous studies used suppression subtractive hybridization (SSH) to identify four genes that are involved in capsaicin metabolism. A BLAST search determined that these genes had high homology with *pAMT, Kas, Acyltransferase* (*AT*), and *FAO*^13^. They are specifically expressed in placental tissue and are accompanied by capsaicin accumulation. Liu *et al.* detected 52 candidate genes including *PAT, TD* and *DHAD* in the CapCyc model by transcriptome sequencing^14^. Qin and Kim detected 51 and 44 of these 52 genes, respectively, in chili genome-wide association studies. Partial genes have been used to study expression characteristics, polymorphisms, and phylogeny^8^,^9^.

Biological and morphological changes in resource utilization and allocation in tissues during development are accompanied by differences in gene expression patterns at various stages of fruit development^14^. In this paper, we illustrate a proposed pathway of capsaicin biosynthesis and analyze the expression of key genes involved in the biosynthetic pathway. The RNA-Seq platform^15^,^16^ was used to analyze the expression profiles of putative capsaicin biosynthetic genes in placental tissue, during five developmental stages of pepper fruit. The identified candidate genes could help to elucidate the molecular basis of pungency.

## Methods

### Plant material and cDNA library construction

The pepper seeds were obtained from “Hainan University, Haikou, China”. Fruits used in the experiments were grown in a growth room at 26 °C/20 °C (day/night temperatures) in 14 h light and 10 h dark per day. Individual flowers were tagged at anthesis. Fruits were randomly collected from different plants at 10, 20, 30, 40 and 50 days after pollination (DAP). Placental tissue (500 mg) from ten individual fruits for each time point were used for RNA extraction. The process was repeated in two independent biological replicates at each developmental stage.

Total RNA was isolated using the Total RNA Isolation System (Takara, Dalian, China) according to the manufacturer’s instructions. RNA quality was checked using an Agilent 2100 Bioanalyzer RNA Nano chip device (Agilent, Santa Clara, CA, USA), and total RNA concentrations were determined using a NanoDrop ND-1000 spectrophotometer (Nano-Drop, Wilmington, DE, USA). RNA with an OD260/280 between 1.8 and 2.2 and an OD260/230 ≥ 1.8 was used for the construction of cDNA libraries.

### Determination of capsaicin content in fruit placenta

Placental tissues of fruits were harvested at 10, 20, 30, 40 and 50 DAP, dried at 50 °C for 6 h, then homogenized with neutralized glass powder (100 mg) using a mortar and pestle. The capsaicinoid extraction from the dried fruits followed the methods proposed by Alothman *et al.* (2012) with slight modifications. Dried pepper samples (0.2 g) were added to ethanol (70%, 2.0 ml) in a 10 mL glass bottle equipped with a Teflon-lined lid. Bottles were capped and placed in a water bath at 80 °C for 4 hours. The samples were sonicated for 50 minutes with a working frequency of 35 kHz. The extract was centrifuged at 12,000 × *g* for 15 min, and the supernatants were filtrated through a 0.45 μm syringe-mounted membrane filter. Samples were stored at −20 °C. Capsaicin analyses were performed by reversed-phase HPLC using a Kromasil Eternity-5-C18 column (4.6 mm × 250 mm). Samples were eluted using a water:methanol (30:70, v/v) mixture. The detection wavelength was set at 280 nm. The injection volume was 10 μL. All measurements were repeated in triplicate.

### cDNA library construction and sequencing

The mRNA content was concentrated from total RNA using RNase-free DNase I (TaKaRa) and magnetic oligo (dT) beads. The mRNA was mixed with the fragmentation buffer and broken into short fragments (~200 bp long). Then, the first strand of cDNA was synthesized with a random hexamer primer. The second strand was synthesized using the SuperScript Double-Stranded cDNA Synthesis kit (Invitrogen, Camarillo, CA) and was purified via magnetic beads. The ends were repaired and tailed with a single 3′ adenosine. Subsequently, the cDNA fragments were ligated to sequencing adapters. Sequencing was accomplished using an Illumina HiSeq™ 2000 platform according to the manufacturer’s protocols.

### Analysis and mapping of DGE reads

Image data generated by the Illumina HiSeq 2000 system was transformed from base calling data to sequence data. Raw reads were filtered to remove adaptor sequences, low quality tags (tags with unknown nucleotides N > 10%), and reads with more than 50% low quality (≤5) bases. The remaining reads were mapped to the reference genome (http://peppergenome.snu.ac.kr) using SOAPaligner/SOAP2. A maximum of two mismatches was determined to be acceptable for alignment purposes.

For gene expression analysis, expression levels were estimated based on the frequency of individual reads and then normalized to the number of reads per kb per million reads (RPKM). We used a probability ≥ 0.8 and a |log2Ratio| value ≥ 1 as thresholds to determine significant changes in gene expression. Finally, these differentially expressed tags were used for mapping and gene annotation.

### Gene ontology (GO) and KEGG pathway enrichment analysis of differentially expressed genes (DEGs)

The Gene Ontology database (http://www.geneontology.org/) classifies genes into three general categories including “cellular component,” “molecular function” and “biological process”^17^. It can be used to predict the biological function of DEGs after mapping the sequencing reads to reference genes. After annotating expressed genes with GO terms, a GO functional classification was performed with WEGO.

All DEGs were mapped to terms in the KEGG database. Genes with a P-value ≤ 0.05 were considered to be differentially expressed. KEGG was used to graphically represent biological pathways and to display the up- or down-regulation of genes in a pathway.

### qRT-PCR analysis for validating the DEGs

Total RNA was isolated from placental tissue at different developmental stages as described above. Genomic DNA was removed by treating with RNase-free Dnase I, and the first strand of cDNA was synthesized using 1 μg of total RNA with PrimeScript^®^ Reverse Transcriptase (Takara) according to the manufacturer’s instructions.

### Analysis of digital gene expression libraries

To investigate transcriptional changes in “Guijiaowang” at different stages of fruit development, the Illumina HiSeqTM 2000 platform was used to perform high throughput sequencing. Five libraries were constructed from samples that were collected at 10, 20, 30, 40 and 50 DAP. This was repeated twice.

### Differentially expressed genes

At each stage of fruit development, mRNA was purified and cDNA libraries were created from two independent samples of whole fruits. These ten libraries were sequenced using the Illumina Hi SeqTM 2000, which produced a total of 16,870,295 raw reads. After filtering reads for quality, a total of 15,550,468 reads remained with a total length of 2,333 Mb.

## Results

### Changes in the placental capsaicin content during five stages of fruit development in ‘Gujiaowang’

**Figure 1:**
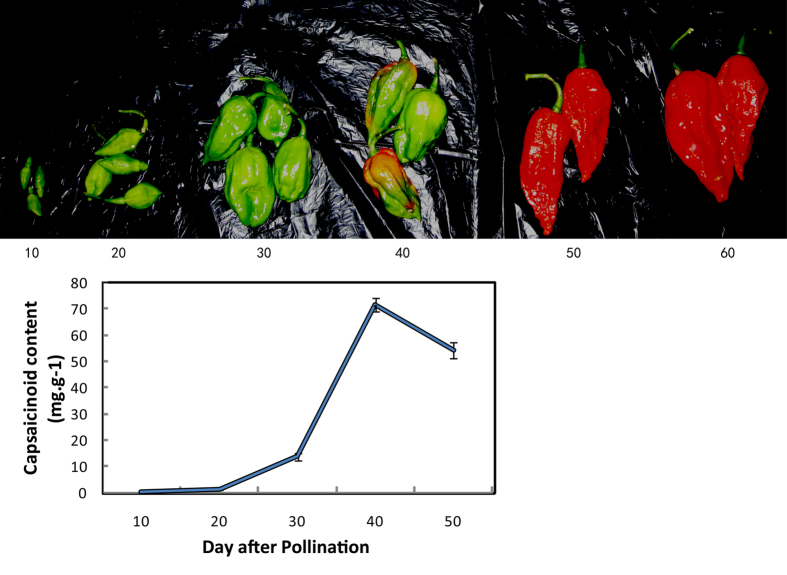
‘Guijiaowang’ fruits and capsaicinoid content of fruit Placenta at different development stage.

Photographs of representative examples from the five stages of fruit development and capsaicin concentrations of chili pepper fruits at the different stages of development are presented in Fig. 1. The results showed limited (0.08 mg/g) capsaicin accumulation in 10 DAP fruit, 1.06 mg/g at 20 DAP, and 13.82 mg/g at 30 DAP. Capsaicin concentrations peaked at about 71.37 mg/g at 40 DAP and then decreased to 54.26 mg/g at 50 DAP. This is consistent with the pattern of capsaicin accumulation described above (Fig. 1).

### Analysis of DGE libraries

Ten libraries were constructed from duplicate samples collected at 10, 20, 30, 40, and 50 days after pollination (DAP). Sequencing of these 10 libraries yielded a total of 225,880,313 clean reads after removing reads that contained an adaptor, Ns, or were of low quality. An overview of the sequencing and alignment statistics are shown in Table 1. The average read length was approximately 49 bases. The number of clean reads per library ranged from approximately 19 million to approximately 26 million. The proportion of clean tags all exceeded 99% for each library (Fig. S1).

**Figure 2:**
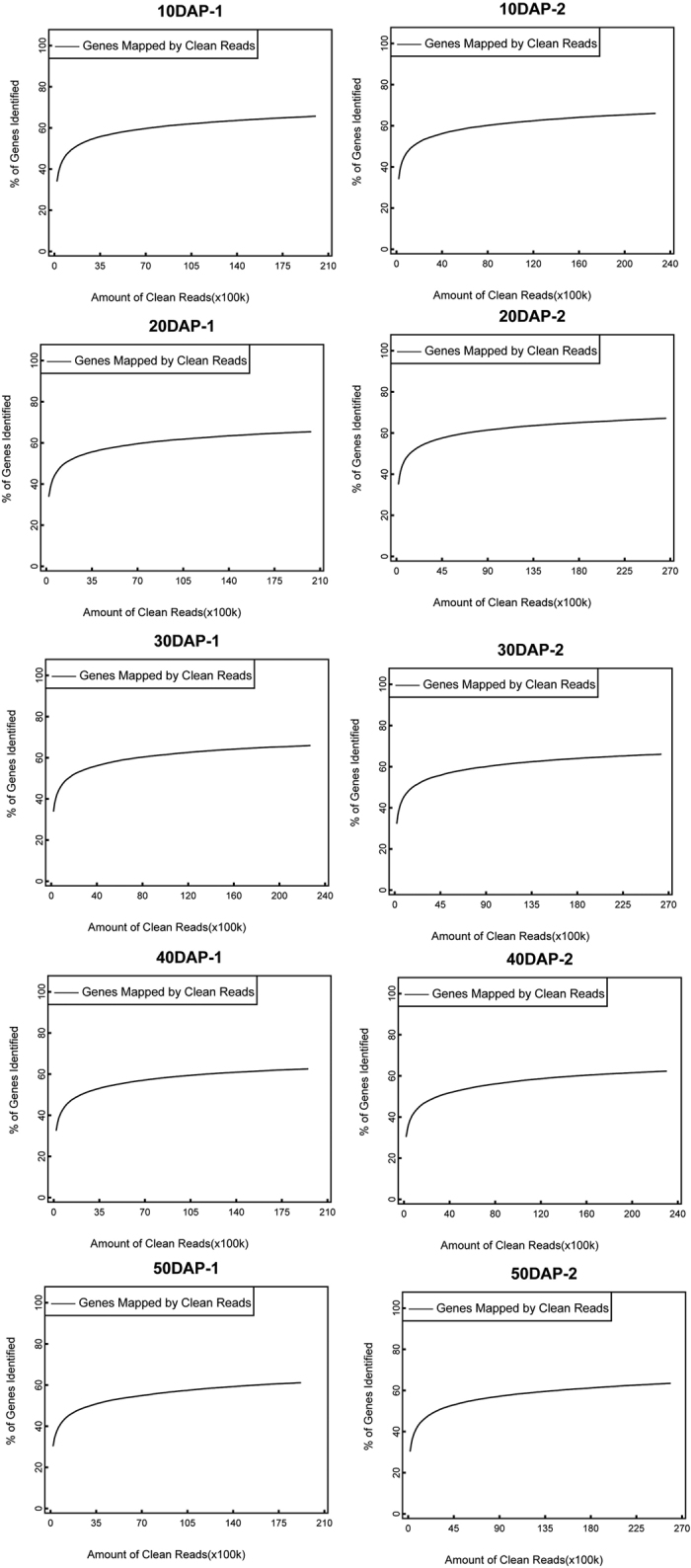
Sequencing saturation analysis of ten sequenced samples.

Saturation analysis was performed to determine whether the sequencing depth was sufficient to detect genes for further analysis. The number of mapped genes increased proportionally with the number of reads (Fig. 2). However, the increase in the number of genes tapered off beyond two million clean reads, implying full saturation of the transcriptome. All of the raw sequence data produced are publicly available (accession number GSE55264).

### Read mapping

The pepper reference genome (http://peppergenome.snu.ac.kr) was used to map the RNA-Seq reads. The proportion of clean reads mapped to the reference genes (Pepper.v.1.55.CDS.fa) in each library ranged from 53.78% and 56.38% (Table 1). Among all reads, 50.14–54.12% per library were uniquely mapped to the pepper reference genome, and 36.45–39.80% of reads were a perfect match to the reference gene.

### Genes detected per subset

**Figure 3:**
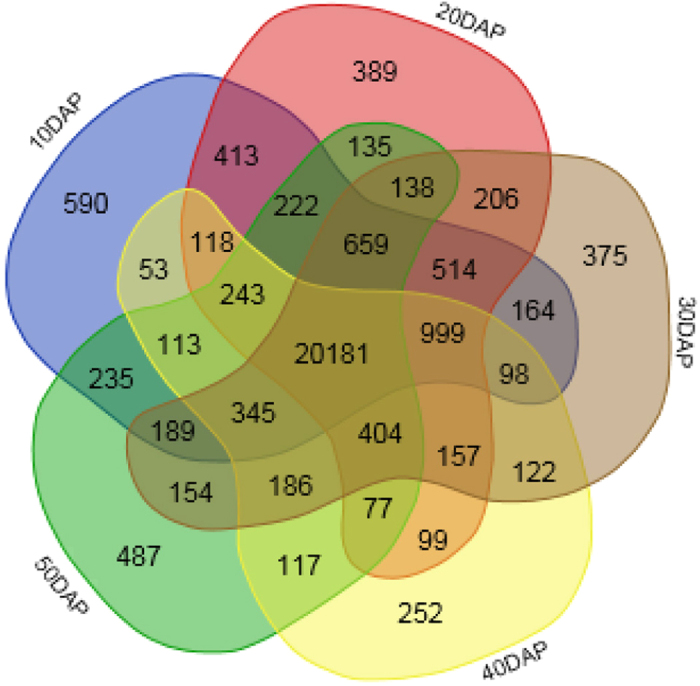
Venn diagram showing the number of gene detected in five different development stages. Each set represent a stage of development. Numbers in each intersection represent the number of genes detected with at least one read (gene tag) in these disjoint sets (intersections).

The number of genes expressed in the five stages of fruit development ranged from 23,564 genes at 40 DAP to 25,137 genes at 10 DAP (Table 2). These values represent the sum of the number of genes derived from two biological replicates at each developmental stage. The total number of genes expressed in at least one of the samples was 28,434. A Venn diagram (Fig. 3) illustrates the intersections between the expressed genes detected at the five developmental stages (10, 20, 30, 40, and 50 DAP). A total of 20,181 genes were expressed in all five stages of development, whereas 2,093 genes were expressed in only one stage. This indicated that while many genes were involved in the overall process of fruit development, far fewer genes were functionally unique to the individual developmental stages of ‘Guijiaowang’ fruit.

### Changes in gene expression profiles during different developmental stages

**Figure 4:**
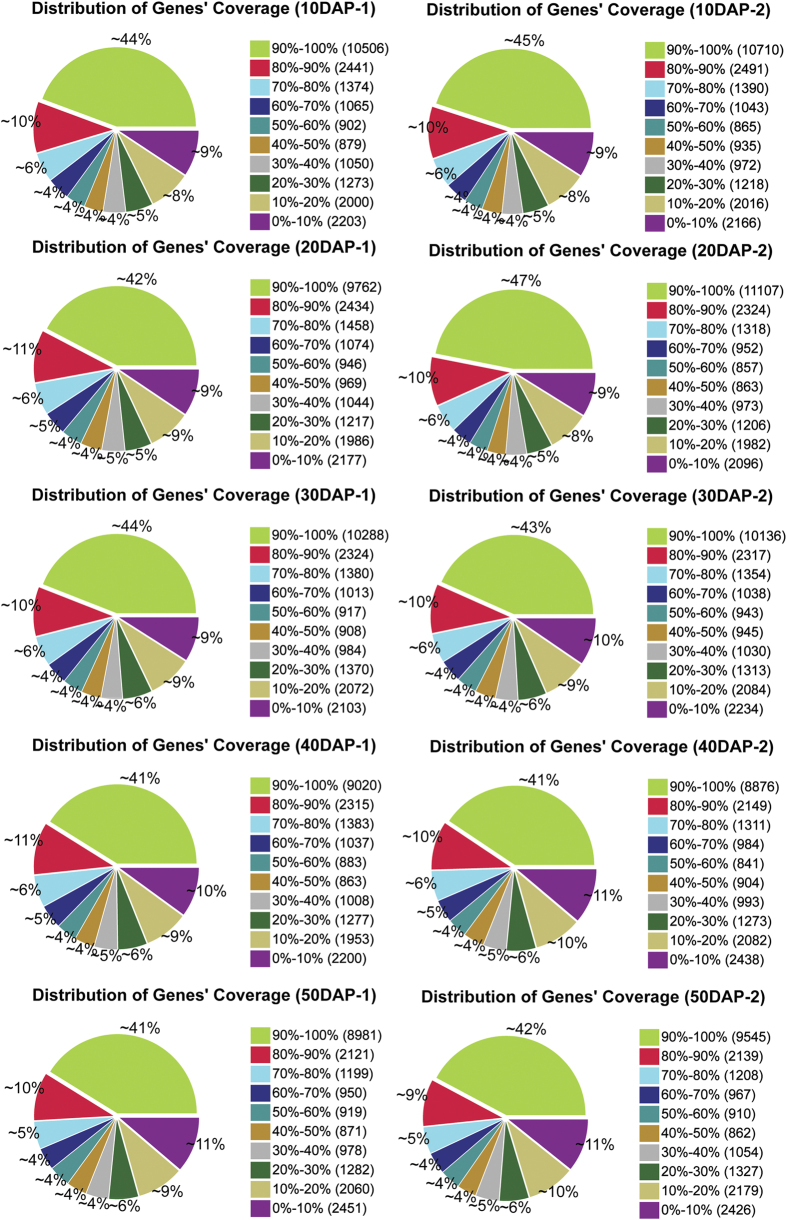
Statistic chart of DEG among the different development stages. The number of up-regulated and down-regulated genes between 10 DAP and 20 DAP; 20 DAP and 30DAP; 30 DAP and 40 DAP; 40DAP AND 50DAP are summarized.

The distribution of a gene’s coverage in each sample was analyzed to evaluate the quality of the RNA-Seq dataset. The term “gene coverage” reflects the proportion of the full gene sequence represented in the RNA-Seq reads (Fig. 4). More than 64.8% of genes had greater than 50% coverage. DGE profile analysis was used to analyze gene expression in the five stages of ‘Guijiaowang’ fruit placental development. The expression level of each gene was calculated using normalized RPKM (Reads Per Kb per Million reads) for each developmental stage^18^.

**Figure 5:**
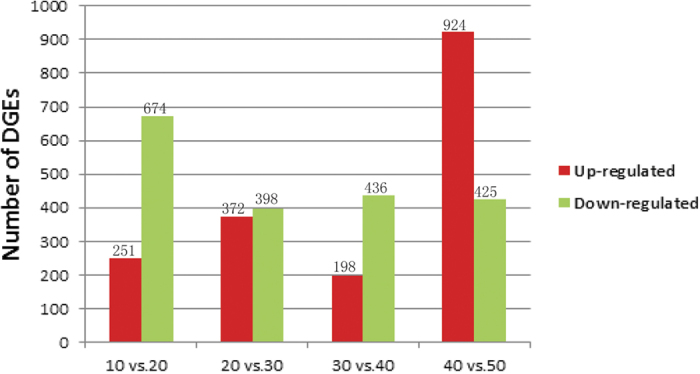
Distribution of genes coverage from ten sequenced samples.

**Figure 6:**
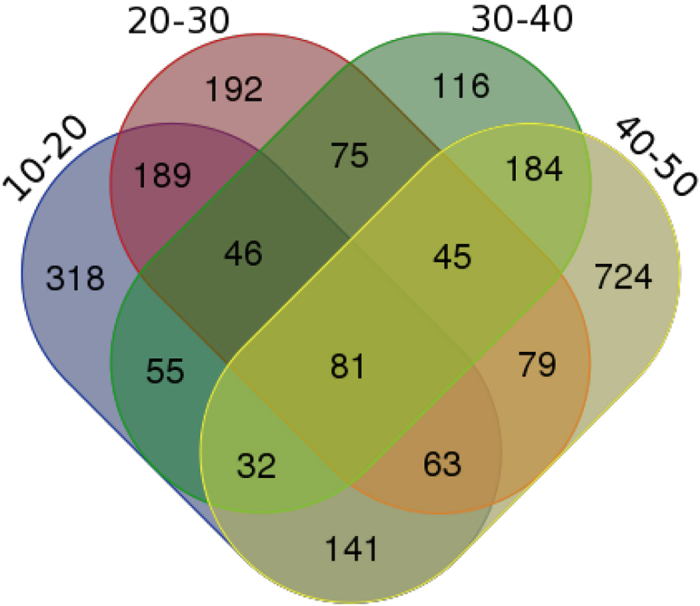
Veen diagram show the number of DGEs identified between consecutive time points.

DEGs between samples were identified using an algorithm developed by Audic *et al.*^19^. The changes in gene expression between consecutive time points was analyzed (Fig. 5). In the early stage of fruit development, 925 genes were differentially expressed between 10 and 20 days. Among these genes, 251 were up-regulated and 674 were down-regulated in 20-day old placental tissue compared with those in 10-day old tissue. Between 20 and 30 DAP, 770 DEGs were observed, with 372 up-regulated and 398 down-regulated. A total of 634 DEGs were detected between 30 and 40 DAP, with 198 up-regulated and 436 down-regulated. Most DEGs were observed between 40 and 50 DAP, with 924 up-regulated and 425 down-regulated relative to the 40 DAP time point. These data are presented in a Venn diagram in Fig. 6. Eighty-one significantly differentially expressed genes were found at different times during placental development. Seventy-five genes were differentially expressed at both 20–30 and 30–40 DAP.

### Functional classification of DEGs in the fruit placenta during different developmental stages

**Figure 7:**
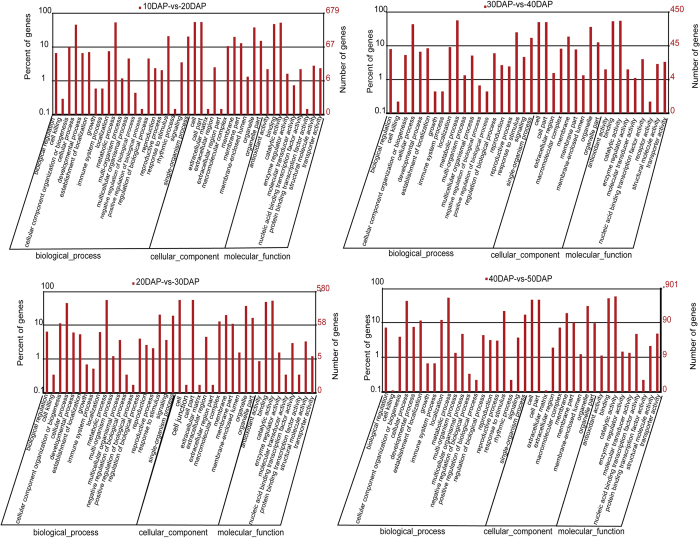
Gene Ontology enrichment analysis of DEGs. Y-axis (right) represents the number of the genes in each category; Y-axis (left) represents the percentage of a specific category of the genes within that main category.

To annotate differentially expressed gene sequences, we performed a BLAST search against the NCBI nonreductant (NR) database (-p blastx -e 1e-5 -m 7), and the results were annotated using Blast2GO and the GO database. For the 10 vs. 20 DAP comparison, of 925 DEGs, 679 genes could be assigned to at least one GO annotation category. The corresponding proportion for the 20 vs. 30 DAP comparison was 580 out of 770; for the 30 vs. 40 DAP comparison, it was 450 of 634; and for the 40 vs. 50 DAP comparison, it was 901 of 1349. Furthermore, for the 10 vs. 20 DAP comparison, 430 genes were categorized as “cellular component,” 534 genes had a molecular function, and 511 genes were involved in a biological process. The respective distributions in the 20 vs. 30 DAP, 30 vs. 40 DAP, and 40 vs. 50 DAP comparisons were 364, 460 and 432, 259, 377, and 342, 506, 734, and 653. Most of these genes were associated with cells, cell parts, extracellular regions, macromolecular complexes, membranes, membrane parts, organelles, and organelle parts. The Gene Ontology significant enrichment analysis of DEGs (10 vs. 20 DAP, 20 vs. 30 DAP, 30 vs. 40 DAP, and 40 vs. 50 DAP) with a P-value cut-off of ≤0.05 is shown in an additional file. In the “cellular component” ontology category, there were 13 and 14 enriched terms in the 10 vs. 20 and 20 vs. 30, comparisons, respectively. No enriched “cellular component” ontology terms were found in the 30 vs. 40 and 40 vs. 50 DAP comparisons (Fig. 7).

In the “molecular function” category, the 10 vs. 20 and 20 vs. 30 DAP comparisons had the most enriched terms (four and six terms, respectively, Fig. 7). Only 39 “protein binding” genes were enriched in the 30 vs. 40 DAP comparison, and 42 “nucleic-acid binding transcription factor activity” genes were enriched in the 40 vs. 50 DAP comparison. In the “biological process” category, “chromosome organization,” “chromatin organization,” “macromolecular complex subunit organization,” “protein-DNA complex subunit organization,” and “nucleosome organization” were significantly enriched in the 10 vs. 20 and 20 vs. 30 DAP comparisons. Only “response to inorganic substance” was significantly enriched in the 30 vs. 40 DAP comparison, and 7 terms were enriched in the 40 vs. 50 DAP comparison (Fig. S2).

We used pathway enrichment analysis on the DEGs in the four sample comparisons (10 vs. 20, 20 vs. 30, 30 vs. 40 and 40 vs. 50 DAP). Only DEGs with a P-value of ≤0.005 were used for the analysis. In the 10 vs. 20 DAP comparison, 35 significantly enriched pathways were detected, including those involved in phenylpropanoid, flavonoid, fatty-acid, and unsaturated fatty-acid biosynthesis; phenylalanine metabolism; and valine, leucine, and isoleucine synthesis and degradation. A total of 28 significantly enriched pathways were detected in the 20 vs. 30 DAP comparison. These included pathways involved in secondary metabolite biosynthesis; alanine, aspartic-acid, glutamic-acid, and pyruvic-acid metabolism; and fatty-acid elongation. A total of 14 significantly enriched pathways existed in the 30 vs. 40 DAP comparison, including secondary metabolite biosynthesis; valine, leucine, and isoleucine synthesis and degradation; and pyruvic-acid and phenylalanine metabolism. A total of 28 significantly enriched pathways existed in the 40 vs. 50 DAP comparison, including secondary-metabolite, phenylpropanoid, and fatty-acid biosynthesis; and pyruvic-acid and phenylalanine metabolism (additional excel file s1).

### Identification of potential capsaicinoid biosynthetic pathway genes

**Figure 8:**
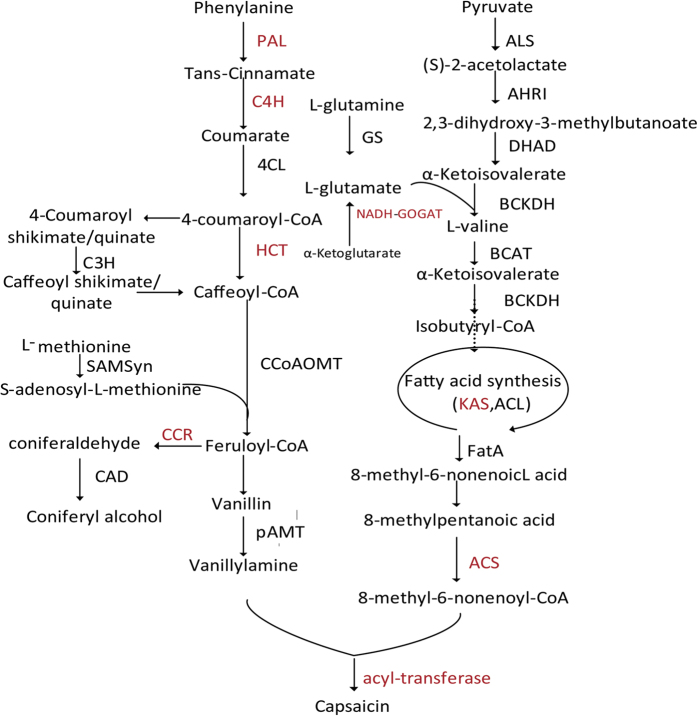
Putative modles of capsaicin biosynthetic pathway.

The capsaicinoid biosynthetic pathway proposed by Mazourek^20^ contains 52 candidate enzymes that are related to capsaicinoid biosynthesis (Fig. 8). Using a BLAST search against the NCBI NR database, 131 genes were annotated as genes involved in the capsaicinoid pathway, except for ferredoxin-dependent glutamate synthase and 3-isopropylmalate dehydrogenase, which were undetected (additional excel file s2). The results showed that many genes encoded the same enzymes. These genes showed different expression patterns, indicating that they may play different regulatory roles in capsaicinoid synthesis.

**Figure 9:**
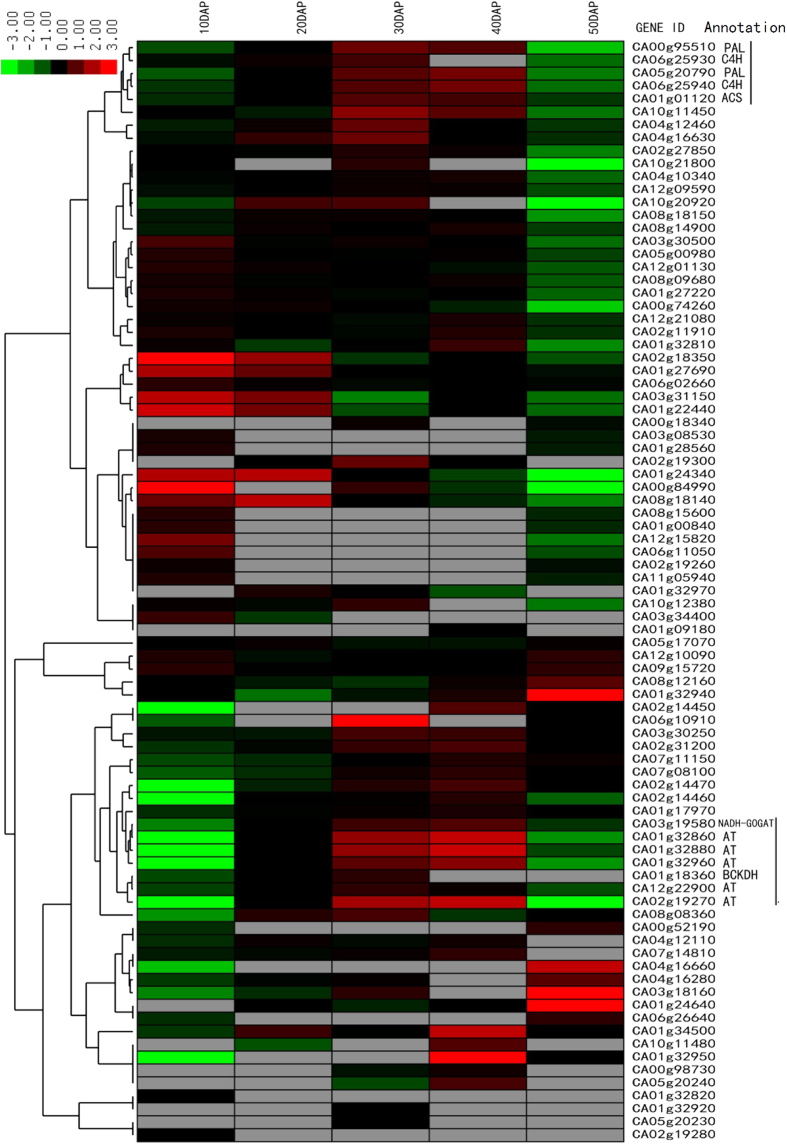
Hierarchical cluster and heat map analysis of 79 capsaicin synthesis genes. Gray bar indicated that the gene expression is too low to detect.

Capsaicin typically accounts for about 70% of the total capsaicinoids. Figure 8 summarizes the main pathway of capsaicin synthesis according to Mazourek *et al.*^20^. It includes phenylpropanoid and benzenoid metabolism, as well as medium-length, branched-chain fatty acid biosynthesis and L-valine metabolism. A total of 79 genes are predicted to encode enzymes|involved in capsaicin biosynthesis. To understand the expression patterns of these genes, the expression patterns were clustered (Fig. 9). We found two groups of genes that clustered together and whose expression patterns were correlated with capsaicin accumulation. These included: *PAL, C4H, Acyl-CoA synthetase* (*ACS*), *NADH-GOGAT, BCKDH,* and *AT*. These genes may play important roles in capsaicin accumulation. This finding was consistent with that of Kim *et al.*^8^. Other genes showed different expression patterns; for example, gene expression in group 3 was higher than gene expression in the aforementioned groups at the other time points.

### Phylogenetic analysis of AT genes and expression variation

**Figure 10:**
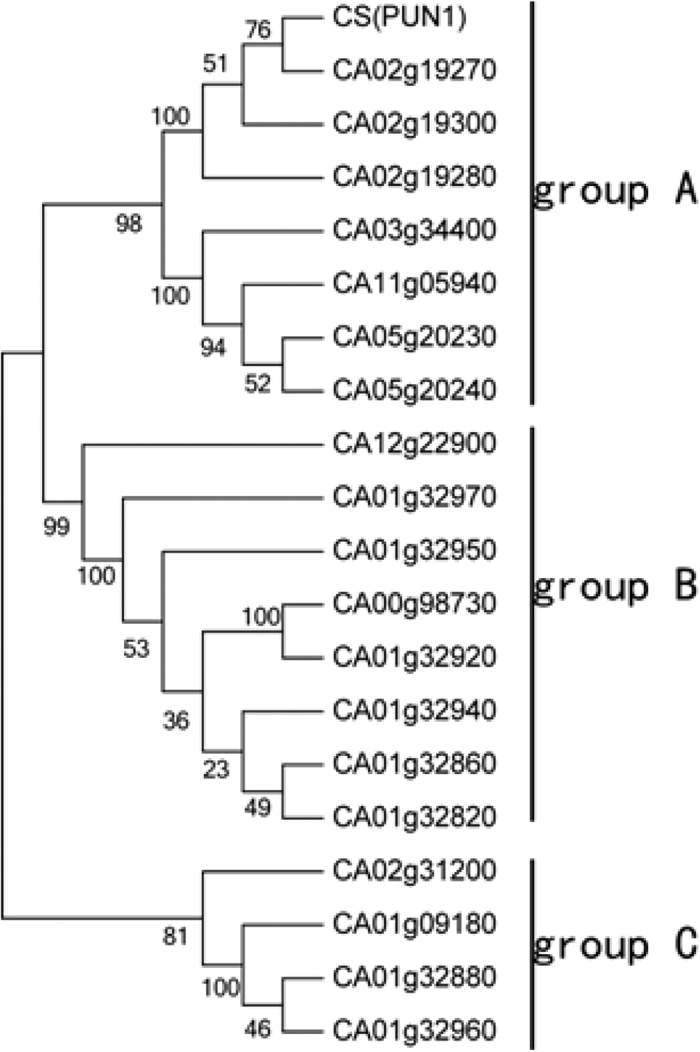
Molecular phylogenetic tree of the amino acid sequences of 21 acyltransferase genes.

Pungent gene 1 (Pun1) proteins encoded by *AT3* are believed to catalyze the last steps in the capsaicin biosynthetic pathway^7^. A total of 21 genes were annotated as putative acyltransferases in our study, including *AT3*. The expression patterns of these differed between the developmental stages. To examine the relationships among them, a phylogenetic tree was constructed using the amino acid sequences of the 21 genes (Fig. 10). All genes were classified into three groups. Group A contained *pun1*; group C had the fewest genes, and three of them had expression patterns that paralleled capsaicin content.

### Clustering analysis of DEGs during placental development

To identify new genes that could contribute to capsaicin accumulation, we examined differences in DEG expression patterns by comparing various combinations of samples (10 vs. 20, 20 vs. 30, 30 vs. 40, 40 vs. 50, 10 vs. 30 and 20 vs. 40 DAP). Based on capsaicin accumulation patterns, we identified 20 new candidate genes expressed in the placenta of ‘Guijiaowang’ fruit. All of these genes were up-regulated in the 20–40 day interval, and then expression significantly declined from 40–50 days (Table 3). This included genes involved in the metabolic pathways of phenylpropanoid biosynthesis, phenylacetic acid degradation, phenylalanine degradation, and glutathione metabolism. This is the first time that these genes have been implicated in capsaicin synthesis and accumulation.

### Quantitative real-time PCR (qPCR) validation

**Figure 11:**
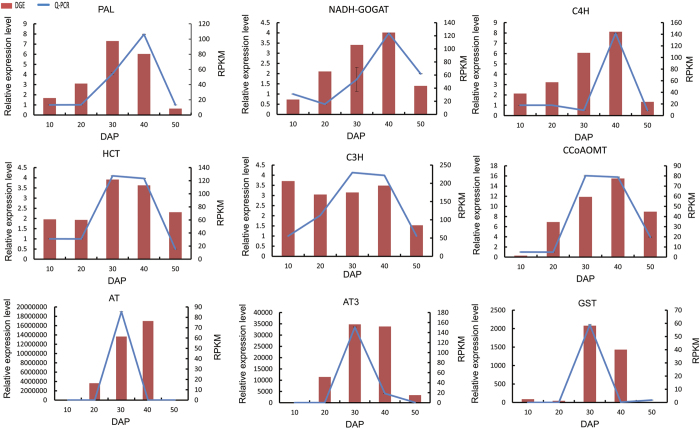
Real-time quantitative validation of digital genes expression data.

To confirm the gene expression levels detected by RNA-Seq, nine randomly selected candidate genes were evaluated using qPCR. The actin gene was used as an internal control (Table 4). The results of the qPCR analyses supported the RNA-Seq analysis (Fig. 11).

## Discussion

Previous studies have indicated that the *Pun1* locus, which is required for the presence of capsaicinoids in pepper, encodes a putative acyltransferase^7^,^11^. Quantitative traits that control developmental processes in pepper are influenced by many genes with small effects. The present study detected 135 known capsaicinoid synthetase-coding genes, but not ferredoxin-dependent glutamate synthase and 3-isopropylmalate dehydrogenase. We identified 20 new candidate genes and reported their involvement in capsaicin synthesis and accumulation for the first time.

AT catalyzes the final step in the capsaicin-biosynthetic pathway, which is one of the most important steps in the pathway^21^. Among the 21 detected acyltransferase-coding genes, *CA01g32960* and *CA02g19270* had expression patterns that paralleled capsaicin accumulation. Their levels of expression at 20–40 DAP were significantly different from those at 10 and 20 DAP. They may have a regulatory effect on the synthesis and content of capsaicin. The *CA01g32970* gene was specifically expressed from 20–40 days and may play an important role in the regulation of capsaicin synthesis.

The production of cinnamic acid from phenylalanine is catalyzed by PAL. PAL formation and accumulation are involved in capsaicin biosynthesis^22^. Five PAL-coding genes were detected, and the expression of *CA00g95510* and *CA05g20790* were found to be positively correlated with capsaicin accumulation, suggesting that these genes play a significant role in the capsaicin synthesis pathway and contribute to capsaicinoid accumulation.

Ferulic acid as a phenylpropanoid intermediate that is produced from caffeoyl-CoA by the action of the enzyme caffeoyl-CoA 3-O-methyltransferase (CCoAOMT)^12^. The increased expression of *CCoAOMT* also increased capsaicinoid accumulation. This study detected five CCoAOMT-coding genes and showed that the expression of *CA02g14470* was positively correlated with capsaicin accumulation.

pAMT is the enzyme that catalyzes the final step in the phenylpropanoid pathway, in which vanillylamine is formed from vanillin^23^. Decreasing or silencing *pAMT* gene expression leads to a significant reduction in capsaicin content in placental tissue^10^. However, no definitive correlation was observed between changes in capsaicin content and the expression of two *pAMT* genes. Therefore, additional experiments will be required to fully understand the functions of the pAMT genes.

Numerous enzymes are involved in the branched fatty-acid synthesis pathway, including ketoacyl-ACP synthase I (KAS), malonyl CoA-ACP transacylase (MCAT), acyl carrier protein (ACL), ketoacyl-ACP reductase (KR), hydroxyacyl-ACP dehydratase (DH), enoyl-ACP reductase (ENR), and thioesterase (TE)^24^. Studies have shown that the fatty-acid synthase genes *Acl1, FatA,* and *Kas* are differentially expressed in *Capsicum* fruits and that the transcript levels of the three genes are positively correlated with the degree of pungency. We detected 11 Kas-coding genes. The expression of two (*CA07g11150* and *CA03g35350*) of these was correlated with capsaicin accumulation, suggesting that these genes may be involved in regulating capsaicin content.

ACS is the enzyme that catalyzes the final step in the branched-chain fatty-acid pathway, in which 8-methyl-6-nonenoyl-CoA is formed from 8-methylpentanoic acid^25^. A total of 11 ACS-coding genes were detected. Three genes (*CA01g01120*, *CA04g10340*, and *CA07g08100*) had expression patterns that were related to capsaicin accumulation. These genes may play crucial roles in the regulation of capsaicin synthesis.

Among the 20 new candidate genes, five genes (*CA06g15270*, *CA00g86910*, *CA10g13930*, *CA04g13110*, and *CA02g04610*) are involved in the known capsaicin biosynthetic pathway. These genes can be used to further explore the regulation of capsaicin synthesis.

## Conclusions

In this study, expressed unigenes involved in secondary metabolism in the placenta of ‘Guijiaowang’ pepper fruit were identified using DGE. Candidate enzyme-encoding genes were identified and subsequently analyzed for expression patterns and phylogenetic relationships. The identified genes confirmed the complexity of the regulatory network thought to be involved in capsaicinoid biosynthesis. The genes identified in this study provide potential targets for controlling the production of capsaicinoid in *Capsicum* breeding programs.

## Additional Information

**How to cite this article**: Zhang, Z.-X. *et al.* Discovery of putative capsaicin biosynthetic genes by RNA-Seq and digital gene expression analysis of pepper. *Sci. Rep.* **6**, 34121; doi: 10.1038/srep34121 (2016).

## Supplementary Material

Supplementary Information

Supplementary Dataset S1

Supplementary Dataset S2

## Figures and Tables

**Table 1:** Summary of read mapping.

Sample ID	Total Reads	Total Base Pairs	Total Mapped	Perfect Match	<=2 bp Mismatch	Unique Match	Multi-positionMatch	Total UnmappedReads
10DAP-1	20,002,310	980,113,190	11,056,527	7,793,992	3,262,535	10,612,172	444,355	8,945,783
	(100.00%)	(100.00%)	(55.28%)	(38.97%)	(16.31%)	(53.05%)	(2.22%)	(44.72%)
10DAP-2	22,664,771	1,110,573,779	12,614,493	8,454,100	4,160,393	12,085,566	528,927	10,050,278
	(100.00%)	(100.00%)	(55.66%)	(37.30%)	(18.36%)	(53.32%)	(2.33%)	(44.34%)
20DAP-1	20,270,262	993,242,838	10,940,658	7,719,242	3,221,416	10,554,431	386,227	9,329,604
	(100.00%)	(100.00%)	(53.97%)	(38.08%)	(15.89%)	(52.07%)	(1.91%)	(46.03%)
20DAP-2	26,549,007	1,300,901,343	14,776,901	9,918,207	4,858,694	14,212,830	564,071	11,772,106
	(100.00%)	(100.00%)	(55.66%)	(37.36%)	(18.30%)	(53.53%)	(2.1 2%)	(44.34%)
30DAP-1	22,672,894	1,110,971,806	1,805,213	8,326,551	3,478,662	11,367,776	437,437	10,867,681
	(100.00%)	(100.00%)	(52.07%)	(36.72%)	(15.34%)	(50.1 4%)	(1.93%)	(47.93%)
30DAP-2	26,246,243	1,286,065,907	14,341,495	9,566,924	4,774,571	13,774,887	566,608	11,904,748
	(100.00%)	(100.00%)	(54.64%)	(36.45%)	(18.19%)	(52.48%)	(2.16%)	(45.36%)
40DAP-1	19,486,774	954,851,926	10,985,869	7,756,014	3,229,855	10,546,443	439,426	8,500,905
	(100.00%)	(100.00%)	(56.38%)	(39.80%)	(16.57%)	(54.1 2%)	(2.25%)	(43.62%)
40DAP-2	22,990,103	1,126,515,047	12,605,059	8,506,821	4,098,238	12,110,211	494,848	10,385,044
	(100.00%)	(100.00%)	(54.83%)	(37.00%)	(17.83%)	(52.68%)	(2.1 5%)	(45.17%)
50DAP-1	19,169,737	939,317,113	10,307,130	7,254,746	3,052,384	9,976,232	330,898	8,862,607
	(100.00%)	(100.00%)	(53.77%)	(37.84%)	(15.92%)	(52.04%)	(1.73%)	(46.23%)
50DAP-2	25,828,212	1,265,582,388	14,090,274	9,488,068	4,602,206	13,587,872	502,402	11,737,938
	(100.00%)	(100.00%)	(54.55%)	(36.74%)	(17.82%)	(52.61%)	(1.95%)	(45.45%)

**Table 2:** Detected number of genes with at least one read per library.

Sample ID	10DAP-1	10DAP-2	20DAP-1	20DAP-2	30DAP-1	30DAP-2	40DAP-1	40DAP-2	50DAP-1	50DAP-2	all
number of genes	2369	23806	23067	23678	23359	23394	21939	21851	21812	22617	28434
union number of genes		25137		24954		24891		23564		23885	

**Table 3:** 20 new candidated genes related to capsaicin synthesis.

GENE ID	Length	10DAP_RPKM	20DAP_RPKM	30DAP_RPKM	40DAP_RPKM	50DAP-1_RPKM	Gene annotation
CA10g05810	153	0.270897565	4.632356898	32.10711615	79.94950869	3.258065086	—
CA06g15270	1329	0.124747411	0	69.05206492	72.28247366	0.440541056	deacetylvindoline O-acetyltransferase-like
CA06g14200	1251	3.50043523	4.214128214	58.73589544	94.95480662	2.130973175	flavin monooxygenase-like protein
CA02g15930	1512	54.61459812	33.68706994	133.6180502	157.7851836	0	probable allantoinase 1-like isoform 1
CA00g86910	429	0.206619196	3.882277667	62.5625232	111.4971353	21.3271175	primary amine oxidase
CA12g06180	1365	0	0	3.111355076	62.78991238	6.689178696	UDP-glucose:glucosyltransferase
CA10g13930	1083	3.146542825	19.44624331	278.2872464	277.5108439	33.72695386	oxidoreductase
CA07g19010	996	2.420185835	0	85.37430203	85.20375455	1.509002015	heterodimeric geranylgeranyl pyrophosphatesynthase small subunit
CA04g13110	1200	6.322755235	22.24629044	22.38486546	133.9452759	0	alcohol dehydrogenase 1-like
CA05g05650	573	1.549090431	0	0	388.6058543	54.8225296	hypothetical protein PRUPE_ppa015802mg
CA04g17720	759	2.36921944	0	0	376.0728045	50.5589510	conserved hypothetical protein
CA02g04610	678	2.441634465	1.235262568	28.20991203	46.09254969	0	glutathione S-transferase parA
CA08g13530	2217	23.94262013	21.88566736	28.89121321	135.5366216	0	ferric reduction oxidase 7
CA01g18250	972	5.857889906	2.000742733	1.035359393	50.71081276	0	bifunctional epoxide hydrolase 2-like
CA01g05600	1131	0.794976815	1.161030877	1.339644492	63.33405326	0	inositol 2-dehydrogenase/D-chiro-inositol 3-dehydrogenase-like
CA03g22430	831	0.049876447	0.042403273	0.227908833	14.87811901	0.482963625	trans-resveratrol di-O-methyltransferase-like
CA04g21310	1053	0.168356382	0.234244861	11.58326028	27.8063755	1.90267745	uncharacterized PKHD-type hydroxylase
CA07g04930	336	0.387163579	3.863877137	31.75933895	104.5324016	4.871828722	—
CA01g11490	690	1.901970416	1.543666686	41.12433866	65.65691205	0.562322006	26.5 kDa heat shock protein
CA02g02940	1251	2.456964293	0	76.42204095	45.73067326	1.748725536	protein ASPARTIC PROTEASE IN GUARD CELL 2-like

**Table 4:** Nucleotide sequences of primers used in real-time quantitative polymerase chain reaction.

Gene ID	Name	Forward primer(5′-3′)	Reverse primer(5′-3′)	Size (bp)
CA04g06670	Actin	TGTTGGACTCTGGTGATGGTGTG	CCGTTCAGCCGTGGTGGTG	164
CA05g20790	PAL	AACTGCTCAACAACAACATTACC	CATCCAACACTTCTCCGTTAGG	150
CA03g19580	NADH-GOGAT	TCCAGTCAGGTTGAAGAAGAGAAG	CAGATTGTGTCGTGAGGAGAGG	187
CA06g25940	C4H	ACGGTGAGCATTGGAGGAAGATG	CCATTCGTCGCAGATTCAGGATTC	148
CA03g30250	HCT	ACTTGTATGGCACGAGGACTAGC	GCAACAGGAGTGGCAGTGAATATC	134
CA08g09680	C3H	GGCAATGGCTGAGGTGATC	GTTGGCGTTGGCTCTGTG	196
CA02g14470	CCoAOMT	ATCGGGAGAACTATGAGATTGGTC	TTGTCCTTGTCAGCGTCCAC	166
CA02g19270	AT1	AGTTCCTTCACCAAGATTCGTAGG	TCCTGAGTCTGCTGACACCTTAG	169
CA01g32960	AT3	CCATCAAACCACCATGTCCATACC	TCAGTCCGTAGGCATCCATCATAG	168
CA02g04610	GST	ACTTTGGTGGTGATGACAATTTGG	GTGAGGATGAGGAAGGGAGTTTG	182
