# Supplementary material for: Discovery of putative capsaicin biosynthetic genes by RNA-Seq and digital gene expression analysis of pepper
Source: Sci Rep. 2016 Oct 19;6:34121. doi: 10.1038/srep34121 (PMC5069471; doi:10.1038/srep34121)
Supplement: Supplementary Information [file srep34121-s1.doc]

**Discovery of putative capsaicin biosynthetic genes by RNA-Seq and digital gene expression analysis of pepper**

Zi-Xin Zhang^1,2,3,4^, Shu-Niu Zhao^2,3^, Gao-Feng Liu^4^, Zu-Mei Huang^2,3^, Zhen-Mu Cao^1^, Shan-Han Cheng^1,2,3*^, Shi-Sen Lin^2,3^

*1.Key Laboratory of Protection and Development Utilization of Tropical Crop Germplasm Resources（Hainan University）, Ministry of Education*

*2.Tropical Crops Genetic Resources Institute, Chinese Academy of Tropical Agricultural Sciences/Key Laboratory of Crop Gene Resources and Germplasm Enhancement in Southern China, Ministry of Agriculture, Danzhou 571737, China*

*3.College of Horticulture and Landscape, Hainan University, Haikou 570228, China*

*4.College of Horticulture, Nanjing Agricultural University, Nanjing 210095, China*

***Corresponding author:**

Shan-Han Cheng, Professor, 165597641@qq.com，*13138939608，*

*College of Horticulture and Landscape, Hainan University, Haikou 570228，China*

*NO.58 Renmin road, Haikou 570228，Hainan Province, China*


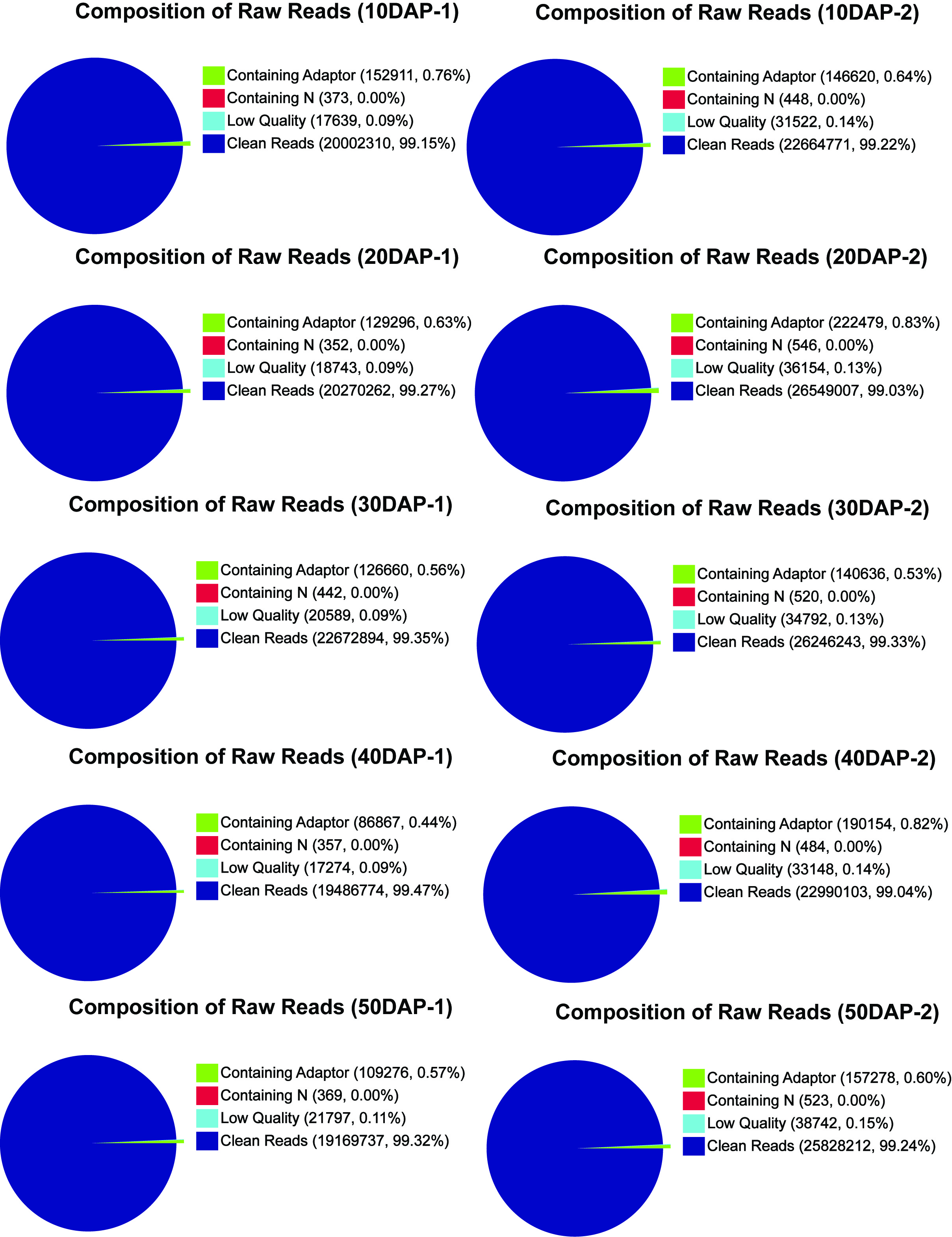
Fig.S1 Distribution of reads from ten sequence samples


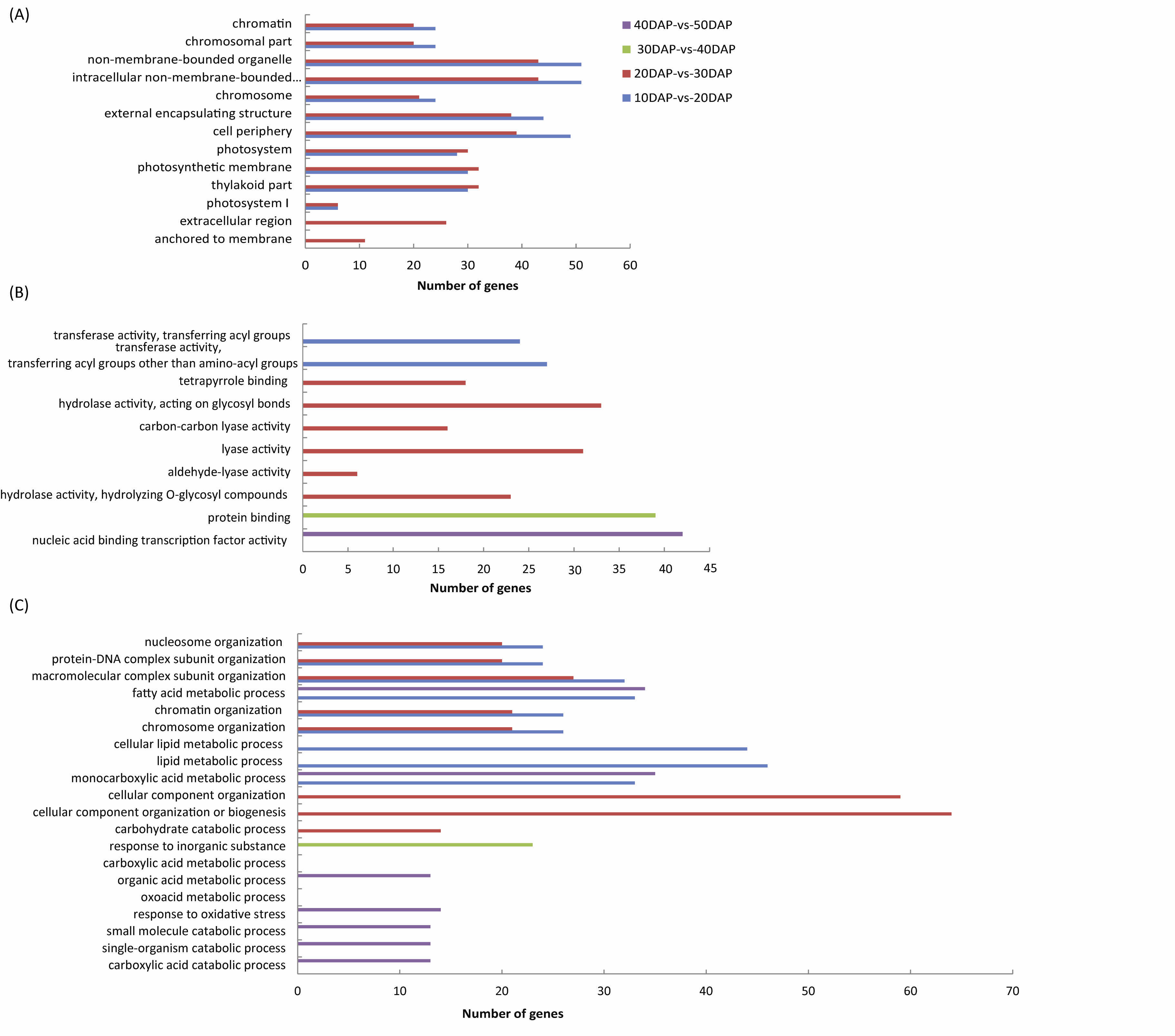


Fig.S2 Function categories of DGEs in the gene ontology
